# CSP I-plus modified rEndostatin inhibits hepatocellular carcinoma metastasis via down-regulation of VEGFA and integrinβ1

**DOI:** 10.1186/s12885-022-10318-8

**Published:** 2022-11-22

**Authors:** Xueqin Chen, Yan Wang, Hancong Liu, Jingjing Zhang, Jie Wang, Xiaobao Jin, Yan Ma

**Affiliations:** 1 Guangdong Provincial Key Laboratory of Pharmaceutical Bioactive Substances, School of Life Sciences and Biopharmaceutics, Guangdong Pharmaceutical University, No. 280, East Waihuan Road, Higher Education Mega Center, Guangzhou, 510006 China; 2 Zhongshan Campus Laboratory Center, Guangdong Pharmaceutical University, Guangzhou, 510006 China

**Keywords:** Hepatocellular carcinoma, Tumor metastasis, rEndostatin, Liver-targeting peptide CSP I-plus

## Abstract

**Background:**

In our previous study, N end of the Circumsporozoite protein (CSP I-plus) modified recombinant human Endostatin (rEndostatin, endostar) (rES-CSP) was constructed, which had antiangiogenic capability and bound to hepatocellular carcinoma in vivo and in vitro. In this study, the inhibition of rES-CSP on hepatocellular carcinoma metastasis was verified in vivo and in vitro, and its possible mechanism was explored.

**Methods:**

Firstly, the impact of rES-CSP on the migration, adhesion of hepatoma cell HCCLM3 was identified by wound healing, transwell, and on metastasis of orthotopic xenograft model was identified in nude mouse. Then the expression of metastasis-associated molecules (MMP2, E-cadherin, integrinβ1) and angiogenesis-related factors (VEGFA) in vitro and in vivo were detected by real-time PCR, western blotting, immunohistochemistry.

**Results:**

Finally, we found that rES-CSP could inhibit the migration and invasion of HCCLM3, and decrease tumor metastasis and growth in nude mouse orthotopic xenograft models. The tumor inhibiting rates of rES-CSP and Endostar were 42.46 ± 5.39% and 11.1 ± 1.88%. The lung metastasis rates of the control, Endostar and rES-CSP were 71, 50, and 42.8%, respectively. Compared with Endostar, rES-CSP significantly down-regulated the expression of VEGFA and integrinβ1. Heparin, a competitive inhibitor of CSP I-plus, which can be bind to the highly-sulfated heparan sulfate proteoglycans (HSPGs) over-expressed in liver and hepatocellular carcinoma, alleviated the down-regulation of VEGFA and integrinβ1.

**Conclusions:**

These indicate that rES-CSP may play a role in inhibiting tumor growth and metastasis by down-regulating the angiogenic factor VEGF and the metastasis-related molecules or by interfering with HSPGs-mediated tumor metastasis.

**Supplementary Information:**

The online version contains supplementary material available at 10.1186/s12885-022-10318-8.

## Introduction

Hepatocellular carcinoma (HCC) accounts for about 85% of all primary liver cancers and is the second leading cause of cancer-related deaths worldwide [1, 2]. Despite significant progress in the diagnosis and treatment of HCC, the 5-year overall survival (OS) rates are 10% for locally advanced and 3% for metastatic disease [3, 4]. High vascularity and frequent metastasis are responsible for rapid recurrence and poor survival of HCC [5]. Therefore, identifying the therapeutic agent or molecular biomarkers that can suppress angiogenesis and metastasis is trend for improving the prognosis and prolonging the survival time of HCC patients [6, 7].

Endostatin (ES) is an endogenous vascular inhibitory factor at the C-terminus of collagen XVIII. Experiments have shown that ES can significantly inhibit tumor blood vessel formation in mice and interfere with many key processes of angiogenesis [8, 9]. Studies have further proved that it can inhibit tumor cells migration, invasion and induce apoptosis by binding to cells with alpha5 integrin, and had anti-tumor activities in vivo through down-regulation of VEGF expression and VEGF actions [10]. Recombinant human Endostatin (rEndostatin, Endostar), by adding MGGSHHHHH to the N-terminus to prolong the half-life of ES, has shown favourable results for tumors combined with chemotherapy or radiotherapy in experimental and clinical research [11–13].

Circumsporozoite protein (CSP), which is the major coat protein on the *Plasmodium* sporozoite, is required for *Plasmodium* sporozoite development and invasion of hepatocytes, and displays highly specific and highly efficient targeting to the liver [14]. CSP I-plus, which is N end of the CSP, could also specifically bind to the liver which overexpress the highly sulfated heparan sulfate proteoglycans (HSPGs) [15]. In addition, the level of HSPGs in hepatocellular carcinoma is significantly different from that in normal liver [16], and is proportional to the metastatic potential of tumor cells, which suggested that HSPGs are important in the differentiation of hepatocellular carcinoma, tumor formation and metastasis [17]. Interfering with the function of HSPGs receptors is a new strategy to inhibit the growth and metastasis of hepatocellular carcinoma [18].

In our previous study, the fusion protein (rES-CSP) was constructed with introducing the CSP I-plus sequence into the C-terminus of Endostar through bioengineering methods, and expressed in *Escherichia coli*. The bioactivity tests turn out that rES-CSP inhibited the proliferation and migration of human umbilical vein endothelial cells (HUVECs) and showed potential antiangiogenic capability on HUVECs tube formation assay and chick embryo chorioallantoic membrane (CAM) assay [19]. Meanwhile, rES-CSP specifically bound to hepatocellular carcinoma and made a direct inhibition in vitro and in vivo [20, 21]. Therefore, we speculate that rES-CSP can inhibit the growth of hepatocellular carcinoma while also suppress the metastasis of hepatocellular carcinoma, and may interfere with the HSPGs receptor-mediated metastasis of hepatocellular carcinoma.

Based on the successful construction of the recombinant protein (rES-CSP) in the early stage, which has the ability to inhibit angiogenesis and could specifically bind to hepatocellular carcinoma, this study will further verify the role of rES-CSP in inhibiting hepatocellular carcinoma metastasis in vitro and in vivo; On the other hand, the underlying mechanism of anti-metastasis will be discussed. The highly metastatic human hepatocellular carcinoma cell line HCCLM3 and nude mouse orthotopic xenograft model were used as the objects. The impact of rES-CSP on the migration, adhesion of hepatoma cell HCCLM3 and metastasis in orthotopic xenograft model was identified. The expression changes of metastasis-associated molecules (MMP2, E-cadherin, integrinβ1) and angiogenesis-related factors (VEGFA) in vitro and in vivo were detected by real-time PCR, western and blotting, immunohistochemistry. This study will provide new experimental evidence and drug development ideas for the treatment of HCC metastasis.

## Materials and methods

### Reagents

Recombinant human endostatin (rEndostatin, endostar) was purchased from Shandong Simcere-Medgenn Bio-Pharmaceutical Co., Ltd. and dissolved in phosphate buffered saline (PBS) at a concentration of 3.0 g/L. CSP I-plus (DNEKLRKPKHKKLKQPADG-NH2) (purity 97.41%) was synthesized by ChinaPeptides in Shanghai and dissolved in PBS at a concentration of 0.3 g/L. CSP I-plus modified rEndostatin (rES-CSP) was prepared after expressing and purification by our laboratory and diluted in PBS at a concentration of 3.0 g/L. Heparin sodium was purchased from Shanghai Aladdin Biochemical Technology Co., Ltd. Reagents were sterilized by passing through a 0.22 μm filter and stored at − 20 °C.

### Animals and cell culture

Female BALB/ C (nu/nu) nude mice weighing 18–22 g were purchased from Guangdong Medical Experimental Animal Center and were reared in an IVC system of the Animal Center of Guangdong Pharmaceutical University (NO. SCXK (Guangdong) 2018–002). The protocol for animal experiments was approved by the Animal Experiment Committee of Guangdong Pharmaceutical University (NO. gdpulac2015036). All procedures were conducted according to the guidelines established by the Institutional Animal Care and Use Committee at the Chinese Academy of Medical Science and the National Institutes of Health. All animal experimental protocol has been carried out in accordance with Arrive guidelines. Human hepatocellular carcinoma cell line HCCLM3 was purchased from the Chinese Type Culture Collection (WuHan). Cells were maintained in Minimum Essential Medium (MEM, Gibco BRL, Rockville, MD, United States) with 10% fetal bovine serum (Life Technologies, Carlsbad, CA, United States), and incubated at 37 °C in humidified atmosphere containing 5% CO_2_. HCCLM3-Luc-GFP stably expressing the luciferase gene (Luc) and Green Fluorescent Protein (GFP) were constructed as follows the patent which was applied by Guangdong Pharmaceutical University [22].

### Cell viability assay

According to the manufacturer’s instructions, cell viability was determined by a Cell Counting Kit-8 (CCK-8). Firstly, 1 × 10^4^ HCCLM3 cells we seeded in 96-well plates. Endostar or rES-CSP (400, 200, 100, 50, 25, 12.5, 6.25, 3.125, 1.56 μg/mL) were then applied for 24, 48, and 72 h. The equimolar CSP I-plus group was administered at a concentration (40, 20, 10, 5, 2.5, 1.25 μg/mL), and the negative control was a drug-free MEM complete medium. After the treatment, the cells were incubated with CCK-8 solution for 2 h. Finally, the absorbance was detected using a microplate reader (DTX880; Beckman Coulter, Inc., Brea, CA) at a wavelength of 450 nm. Experiments were independently repeated three times. According to the inhibition rate, the half-maximal inhibitory concentration (IC_50_) was analyzed using the probability distribution and the probit procedure of SPSS13.0.

### Cell migration assay

Cell migration was firstly assessed using a wound healing assay. HCCLM3 were seeded onto 12-well plates and grown to more than 80% confluence, and scratched with 200 μL pipette tips across the monolayer. Cell debris was removed by washing with PBS. The cells were treated with 10 μg/mL CSP I-plus, 100 μg/mL Endostar, 100 μg/mL rES-CSP. Images were taken at 0, 12, 24 and 48 h under an inverted microscope. The area of wound healing was measured with Image J software. Each experiment was performed in triplicate.

Cell-migration was further tested in transwell chambers of the 24-well plate with a polycarbonate membrane (8 μm pore size, Millipore, Bedford, MA). Cells (2 × 10^5^) in serum-free MEM were added to the upper chamber, and incubated in the absence or presence of endostar, rES-CSP, with addition of 600 μL complete medium containing 20% FBS to the lower chamber. After 24 h, the cells remaining on the upper chamber were removed with cotton wool, whereas the cells that migrated to the bottom of the membrane were fixed with 4% paraformaldehyde (PFA) for 10 min at room temperature, and were stained with 0.1% crystal violet for 10 min. Stained cells were visualised under an inverted microscope (Olympus, Japan), and randomly count the number of cells in 5 non-repeating fields using Image J software. The average was taken to calculate the migration.

### Cell invasion assay

The cell invasion assay was performed using matrigel coated in transwell chamber of the 24-well plate with a polycarbonate membrane (8 μm pore size, Millipore, Bedford, MA). Firstly, basement membrane Matrigel (BD Martrigal™ Basement Membrane Matrix, BD Bioscience) was diluted with serum-free MEM in 1:1, and was used for coating the upper surface of the polycarbonate membrane with 100 μL per well and was solidified for 3 h at room temperature. Then, cell invasion capacity was assessed by the same steps with a Transwell chamber mentioned above in cell migration assay. The invaded cells, which penetrate the matrigel to the bottom of the membrane, were photoed and count.

### Animal model, groups and administration

The orthotopic xenograft model of HCC was established by cell-suspension injected in liver of nude mice. HCCLM3-Luc-GFP cells in logarithmic growth period were digested and resuspended in the serum-free MEM, and adjusted the concentration to 5 × 10^7^cells/mL. The 6 to 8-week old female BLAB/c nude mice were anaesthetized by the pentobarbital sodium (50 mg/kg) and its abdomen were opened to expose the liver. Then 20 μL cell-suspension was implanted slowly into the liver using the 28G aseptic micro injector. The liver was pressed with the sterile gauze until bleeding stopped and sent back to the abdomen, and the abdominal wall was sutured. All procedures used in this method should be operated under the sterile condition. After the operation, the mice were raised in the SPF condition and the mice postoperative situation was observed every day. One week later, mice were randomly divided into three groups (*n* = 8 mice in each group), and received tail vein injection with 15 mg/kg Endostar, 15 mg/kg rES-CSP, or with an equal volume of normal saline (NS), once every 2 day, for 30 days.

### Evaluation of the tumor growth and its metastasis in nude mice model by in vivo and ex vivo imaging

For in vivo imaging, mice were intraperitoneal administered the luciferase substrate D-luciferin (150 mg/kg), and anesthetized with a dose of 50 mg/kg pentobarbital sodium on days 7, 14, 21 and 28 d after orthotopic implantation. Growth and metastasis of the tumor were monitored on the Tanon 5200Multi imaging system (Tanon Science & Technology Co. Ltd.). After the last in vivo imaging (28 day), the mice were sacrificed, and their major organs (heart, liver, spleen, lung, kidney) and tumors were harvested for ex vivo imaging. The intensity of the luminescence were analyzed by Image J software.

### Observation of the tumor and its lung metastasis in nude mice model by histopathology

The nude mice were sacrificed after the last in vivo imaging, their heart, liver, spleen, lung, kidney and tumor were respectively harvested. Tumors were measured, weighed and photoed. Tumor volume was estimated by the formula *V*_*tumor*_ = a^2^ × b /2, where a was the short and b was the long tumor axis. And tumor volume were used to evaluate the anti-tumor effects. Inhibition rates (%) = [*V*_tumor(control)_ - *V*_tumor(treated)_] / *V*_tumor(control)_. The lung tissues of the nude mice were fixed by the Bouin’s fluid (75 mL 0.9–1.2% picric acid, 5 mL glacial acetic acid and 25 mL 40% formaldehyde) for 24 h, and soaked with anhydrous ethanol for 12 h. The metastatic tumor foci in lung were photographed and observed.

#### RNA isolation and real-time polymerase chain reaction (real-time PCR)

HCCLM3 were treated with 10 μg/mL CSP I-plus, 100 μg/mL Endostar, 100 μg/mL rES-CSP, rES-CSP + 50 μg/mL sodium heparin for 48 h. The cells were harvested, and total RNA was extracted using RNA simple Total RNA Kit (DP419, TIANGEN, Beijing, China). First-strand cDNA was synthesized using 2 μg total RNA primed with oligo (dT) as described in PeimeScriptreagent Kit (RR037A, TaKaRa, Japan). Real-Time PCR was performed in Fast Real-Time PCR system (CFXTM, Bio-Rad, USA) using Power SYBR Premix Ex Taq II (RR820A, TaKaRa, Japan) in a 20 μL reaction volume. All specific primers are listed in Table 1. Cycling parameters were optimized as follows: pre-denaturation 95 °C for 1 min, 40 cycles of 95 °C for 10s, 60 °C for 30s. The amplicons were quantified using the comparative threshold cycle method and expression was shown relative to GAPDH.

**Table 1:** Primer sequence used in real-time PCR

Primer name	Primer sequence (5′–3′)
VEGF-F	TCTGCTGTCTTGGGTGCATT
VEGF-R	AGCTGCGCTGATAGACATCC
MMP2-F	TGATGGCATCGCTCAGATCC
MMP2-R	GGCCTCGTATACCGCATCAA
E-cadherin-F	AGGCCAAGCAGCAGTACATT
E-cadherin-R	AAATGTGTCTGGCTCCTGGG
Integrinβ1-F	GTCGTGTGTGTGAGTGCAAC
Integrinβ1-R	CCAAGGCAGGTCTGACACAT
GAPDH-F	GGTGAAGGTCGGAGTCAACGG
GAPDH-R	CCTGGAAGATGGTGATGGGATT

### Western blotting

The cells treated as above were collected and lysed in cell lysis buffer (P0013, Beyotime, Shanghai, China) supplemented with PMSF protease inhibitors (Thermo Fisher Scientific, USA). Protein concentration of cell extracts was determined using BCA Protein Assay Kit (P0012, Beyotime, Shanghai, China). Whole-cell extracts (~ 35 μg/lane) were denatured and resolved on 10% SDS-PAGE, and then electrotransferred onto a polyvinylidene difluoride (PVDF) membrane (Millipore). Non-specific proteins on the membranes were blocked with 5% of nonfat milk (BD) in TBST. The membranes were then probed with respective primary antibodies against VEGFA (ab52917), MMP2 (ab37150), integrinβ1 (ab30394), and E-cadherin (ab76055) procured from Abcam, β-actin (AA128) from Beyotime Biotechnology, Shanghai, China. The membranes were washed and incubated with the horseradish peroxidase (HRP)-conjugated secondary antibodies (A0216 and A0208, Beyotime, Shanghai, China). The protein bands were visualized using an enhanced chemiluminescence detection kit (P0018AS) on the Tanon 5200Multi imaging system, and quantified using Image J software.

### Immunohistochemistry

Briefly, tumor sections were dewaxed and rehydrated according to standard protocols, and immersed in 10 mmol/L citrate buffer (pH 6.0) and boiled in a pressure-cooker for 10 min. 3% H_2_O_2_ in methanol was preheated at 37 °C, and used to block the endogenous peroxidase in darkness for 15 min. Nonspecific binding was blocked by incubating the sections with 1% BSA in a humid chamber for 30 min. The primary antibodies against VEGFA (ab52917), MMP2 (ab37150), integrinβ1 (ab30394), and E-cadherin (ab76055) were diluted in accordance with the instructions, and incubated at 4 °C overnight. HRP-labeled antibody (A0216 and A0208, Beyotime, China) was used in a dilution 1:50, and incubated at 37 °C for 1 h. The bound antibody was visualized with DAB coloring solution dropwise, and observed under a microscope to stop the reaction with single distilled water. Then counter-stained with hematoxylin, differentiated with 0.1% hydrochloric acid alcohol, rinsed with running water and returned to blue. Images were photographed under light microscope (Olympus, Japan). The positively stained area was analyzed by Image J software.

### Statistics analysis

Data are presented as Mean ± SD and analyzed by SPSS13.0. The difference between two groups was tested with *t*-test. When more than two groups, one-way analysis of variance (ANOVA) was done with Bonferroni’s multiple comparison exact probability test. For all statistical tests, a value of *p* < 0.05 was accepted as statistically significant.

## Results

### rES-CSP inhibits proliferation of HCCLM3 cell

**Fig. 1:**
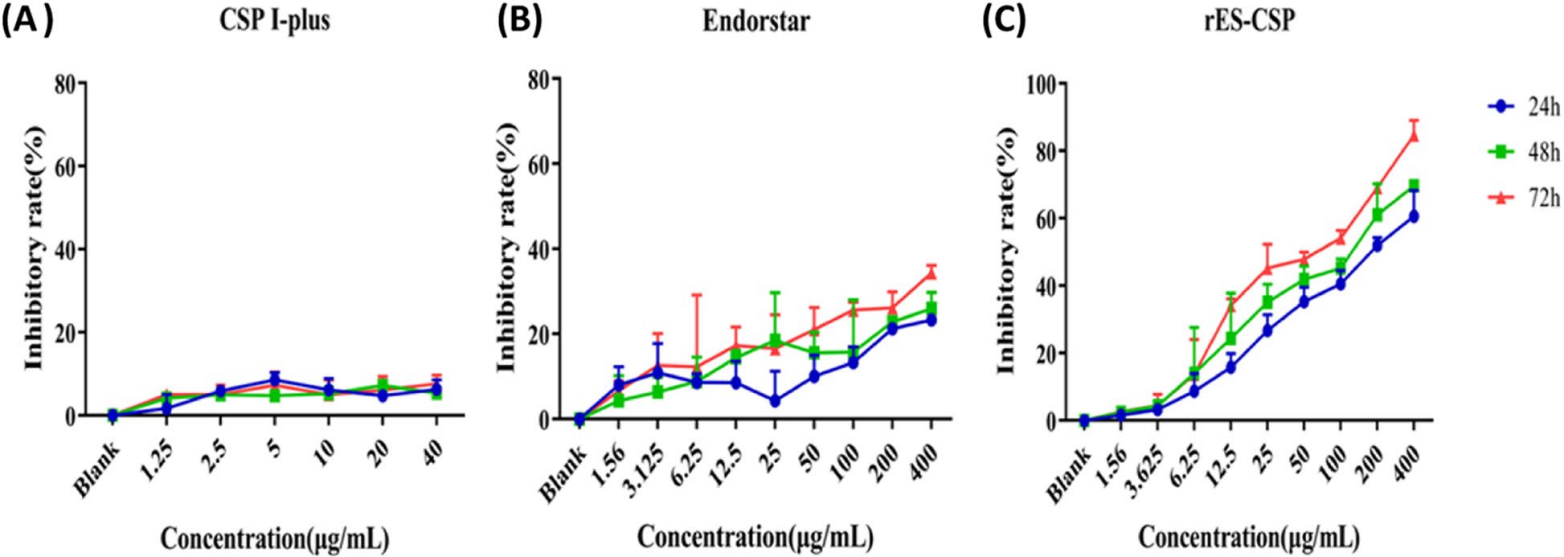
The proliferation of HCCLM3 cells by CCK-8 assay. **A** The inhibition rate of CSP I-plus at a different concentration for 24, 48, and 72 h; **B** The inhibition rate of Endostar at a different concentration for 24, 48, and 72 h; **C** The inhibition rate of rES-CSP at a different concentration for 24, 48, and 72 h

To investigate cell proliferation at different concentration of rES-CSP，cells were treated with rES-CSP, Endostar or CSP I-plus for 24, 48 or 72 h, and cell viability was measured by the CCK-8 assay. As compared with the blank group, rES-CSP inhibited proliferation of hepatoma cells HCCLM3 in a dose and time-dependent manner (Fig. 1). The effect of equimolar Endostar was significantly weaker than rES-CSP, while CSP I-plus had little effect on the proliferation of HCCLM3 cells. The Probit procedure of SPSS13.0 was used to analyze the dose-response relationship to obtain the half-inhibitory concentration (IC_50_). The IC_50_ of rES-CSP were 171.36 ± 34 μg/mL, 99.59 ± 2.65 μg/mL and 55.25 ± 6.68 μg/mL at 24 h, 48 h and 72 h. According to the IC_50_ of rES-CSP at 48 h, 100 μg/mL rES-CSP, the equimolar Endostar 100 μg/mL and 10 μg/mL CSP I-plus were used in cell migration assay and invasion assay.

### rES-CSP inhibits migration and invasion of HCCLM3 cell

**Fig. 2:**
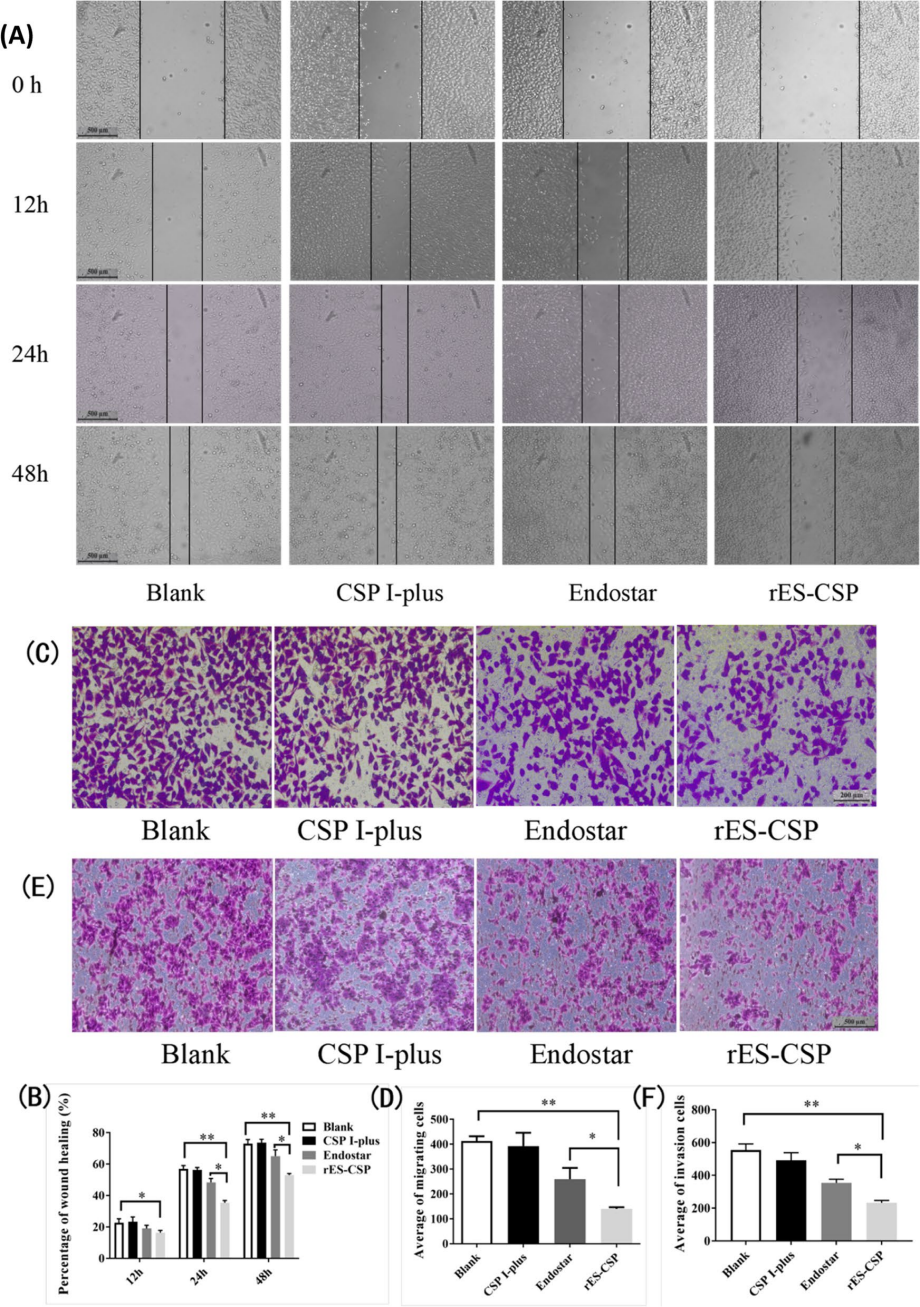
The migration and invasion of HCCLM3 by wound healing and Transwell. **A** Wound healing in HCCLM3 cells; **B** The percent migrated area of wound healing. **C** Crystal violet staining of migrated cells; **D** Statistics of the migrated cells number. **E** Crystal violet staining of invaded cells; **F** Statistics of the invaded cells number. rES-CSP group vs Blank group or Endostar group *P < 0.05, **P < 0.01; *n* = 3

The effect of rES-CSP on cell migration was investigated using the wound healing assay and Transwell. Cell wound gradually healed with time. Cells treated with rES-CSP exhibited closure of wound by 54%, while 65 and 76% in Endostar and blank group at 48 h (Fig. 2A, B). Transwell analysis showed that the number of migrated cells in Endostar or rES-CSP group was significantly decreased compared with the blank group (Fig. 2C, D). These results indicated that rES-CSP could inhibit the migration of HCCLM3 cells, and its effect was stronger than the Endostar (*P* < 0.05).

To assess the impact of rES-CSP on cell invasion, HCCLM3 cells were subjected to Matrigel transwell assays. The number of cells invaded into the chamber in the rES-CSP group was significantly lower than that of the blank group (*P* < 0.01), and it was significantly lower than that of the Endostar group, too (*P* < 0.05) (Fig. 2E, F). Hence, rES-CSP had the effect of inhibiting the invasion of HCCLM3 cells.

### rES-CSP inhibits tumor growth in tumor-bearing nude mice

**Fig. 3:**
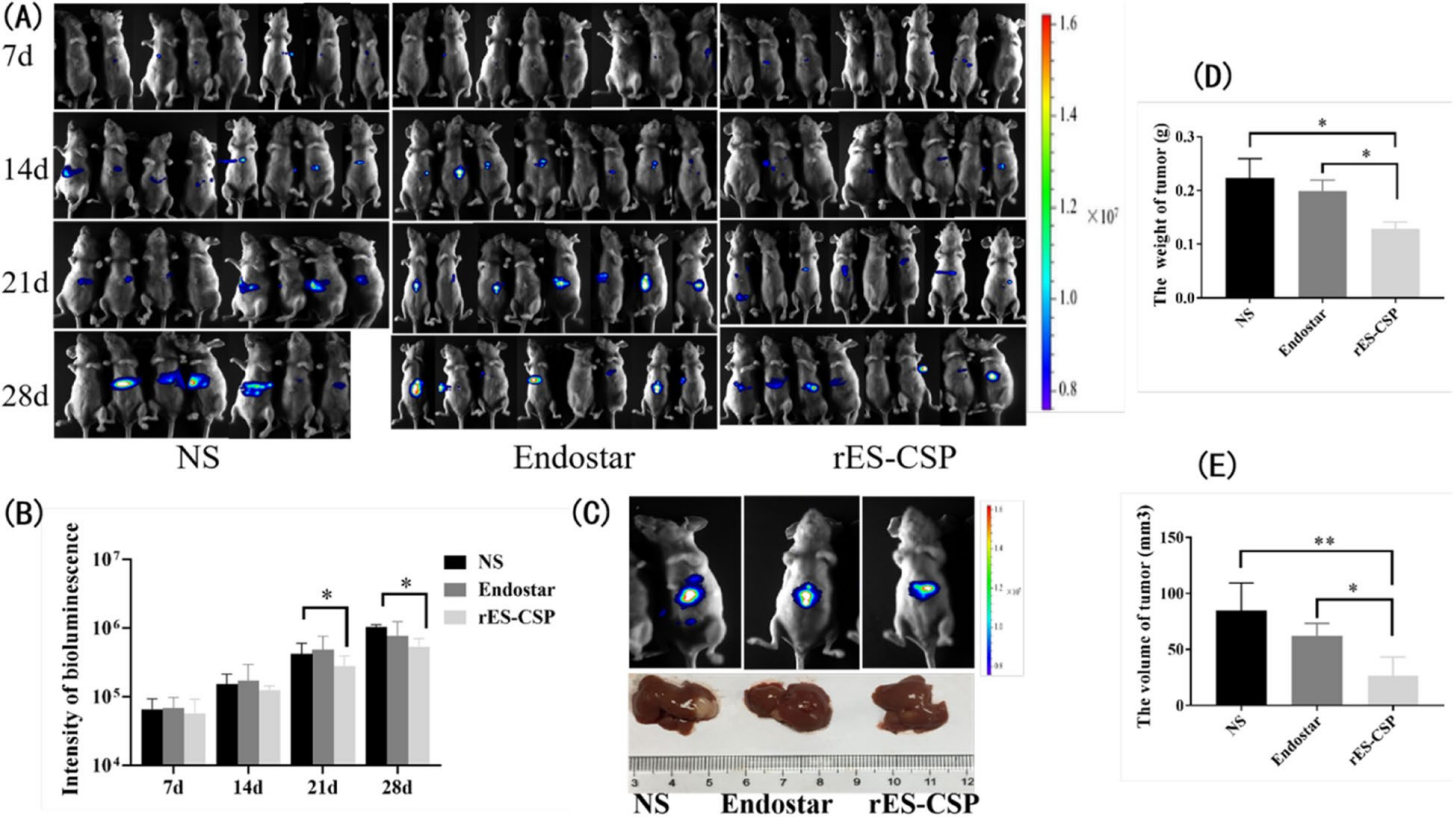
Tumor growth in nude mice bearing orthotopic xenograft tumor. **A** In vivo imaging of orthotopic xenograft tumor; **B** Statistical analysis of bioluminescence intensity; **C** Tumor size of orthotopic xenograft tumor; **D** Statistical analysis of the tumor weight; **E** Statistical analysis of the tumor volume. rES-CSP group vs Blank group or Endostar group *P < 0.05, **P < 0.01; *n* = 6

Dynamic monitoring of tumor growth in nude mice bearing orthotopic xenograft tumor was performed by in vivo imaging. The bioluminescence was detected on the 7th day after implantation, the signal intensity of the three groups gradually increased and expanded. The bioluminescence enhancement intensity of the rES-CSP group was weaker than that of the control group (Fig. 3A). In addition, tumors were weighed and the size of tumors were measured (Fig. 3B, C, D). Compared with the Endostar group and the control group, the rES-CSP group has a statistically significant difference in tumor weight and tumor volume. The inhibition rates of the rES-CSP and Endostar were 42.46 ± 5.39% and 11.1 ± 1.88%, respectively (Fig. 3E). In summary, rES-CSP could inhibit the tumor growth in nude mice bearing orthotopic xenograft tumor.

### rES-CSP inhibits tumor metastasis in tumor-bearing nude mice

**Fig. 4:**
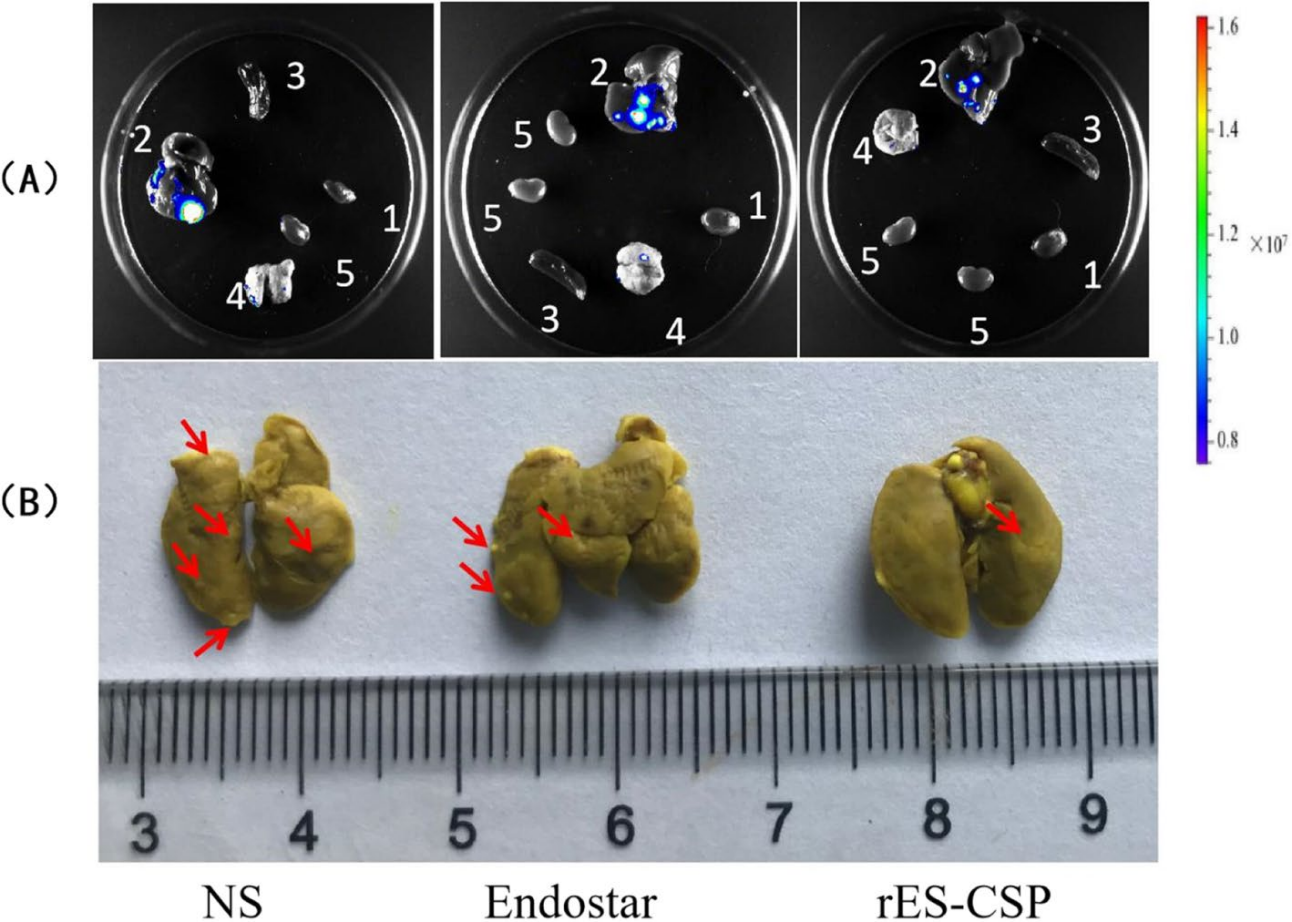
Tumor metastasis in nude mice bearing orthotopic xenograft tumor. **A** The metastasis of orthotopic xenograft tumor by ex vivo imaging; 1: heart; 2: liver; 3: spleen; 4: lung; 5: kidney; **B** The lung metastasis of orthotopic xenograft tumor by Bouin’s taining

In order to track tumor metastasis, the bioluminescence of the heart, liver, spleen, lung and kidney tissues were recorded by ex vivo imaging. The lung tissue has the luminescence signal indicating the presence of metastases in the lungs (Fig. 4A). The metastasis of the rES-CSP group was at the edge of the lung tissue and the intensity of the luminescence was weaker than that of the Endostar group and the saline group. Next, the lung tissue was stained with Bouin’s solution, it was yellow and the tumor was white. Results showed that lung metastasis occurred in orthotopic xenograft nude mice models with HCCLM3 (Fig. 4B). The lung metastasis rates of the NS group, the Endostar group, and the rES-CSP group were 71, 50, and 42.8%, respectively. These confirmed that rES-CSP has the ability to inhibit lung metastasis of hepatocellular carcinoma.

### Effects of rES-CSP on angiogenesis-related factor and metastasis-associated molecule in HCCLM3 cell

**Fig. 5:**
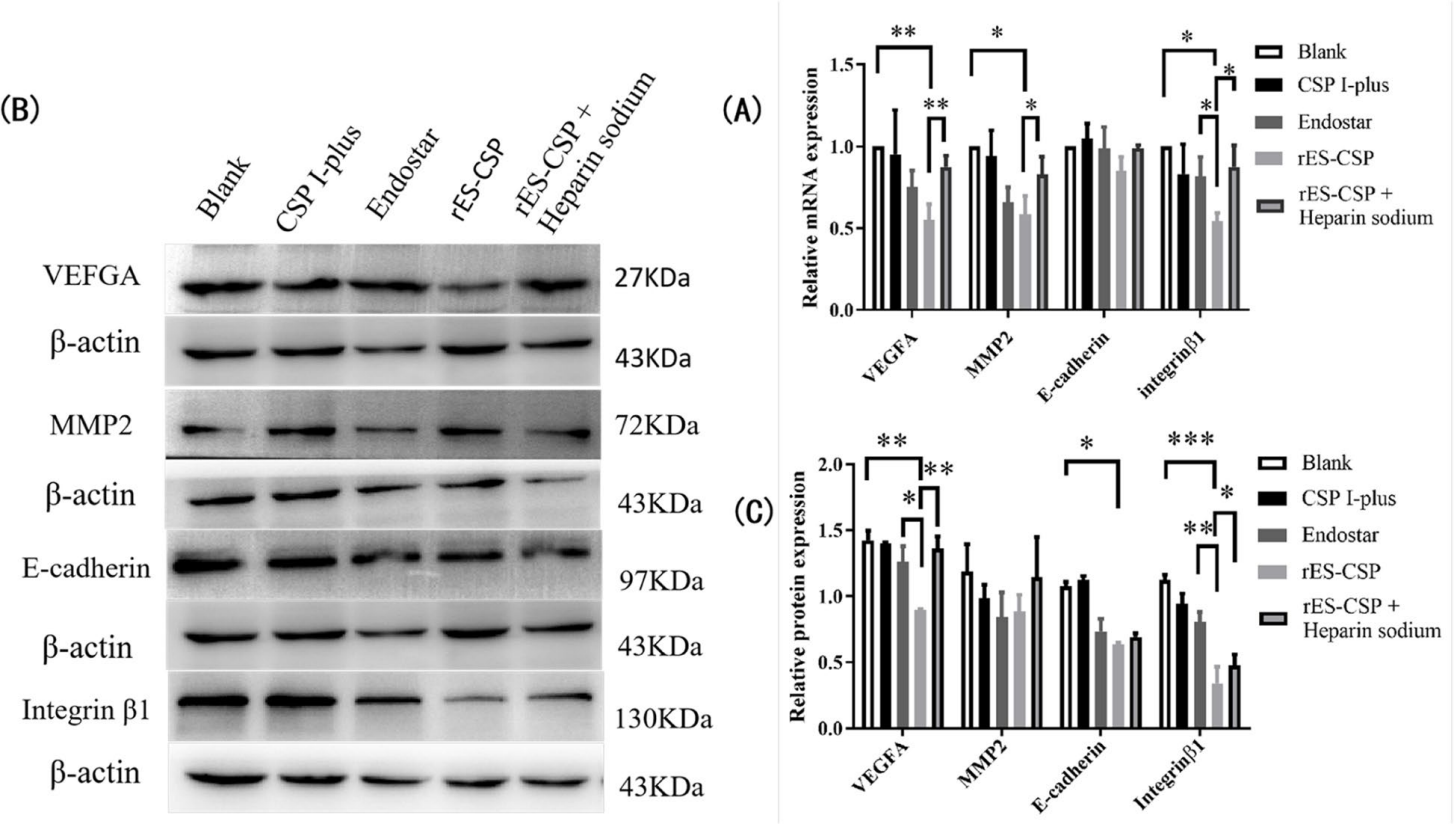
The expression of angiogenesis-related factor and metastasis-associated molecule in HCCLM3 cell. **A** mRNA expression by RT-PCR; The mRNA expression was quantified using the comparative threshold cycle method. mRNA expression of angiogenesis-related factor VEGFA and metastasis-associated molecule MMP2, integrinβ1 and E-cadherin were shown relative to GAPDH. **B** Protein expression by Western blotting (Full-length blots are presented in Supplementary Figures); **C** Statistical analysis of the value of angiogenesis-related factor VEGFA and metastasis-associated molecule MMP2, integrinβ1 and E-cadherin /β-actin. The protein bands were quantified using Image J software, protein expression of VEGFA, MMP2, integrinβ1 and E-cadherin were shown relative to β-actin rES-CSP group vs Blank group; rES-CSP group vs Endostar group; rES-CSP group vs rES-CSP+ heparin sodium group *P < 0.05, **P < 0.01, ***P < 0.001; *n* = 3.

To study the effect of rES-CSP on angiogenesis and tumor metastasis, mRNA and protein level of the angiogenesis-related factor VEGFA and metastasis-associated molecule MMP2, integrinβ1, E-cadherin were analyzed by RT-PCR and Western Blotting in HCCLM3 cell. The results showed that the mRNA and protein level of VEGFA and integrinβ1 in rES-CSP group were significantly lower than the blank group and Endostar groups(*P* < 0.05). In rES-CSP and Heparin sodium group, the level of VEGFA and integrinβ protein was significantly up-regulated compared with the rES-CSP group (Fig. 5). Those showed that rES-CSP could down-regulate the expression of the vascular endothelial growth factor VEGFA and metastasis-associated molecule integrinβ1, and heparin sodium could affect the function of rES-CSP.

### Effects of rES-CSP on angiogenic factor and metastasis-associated molecule in orthotopic xenograft tumor

**Fig. 6:**
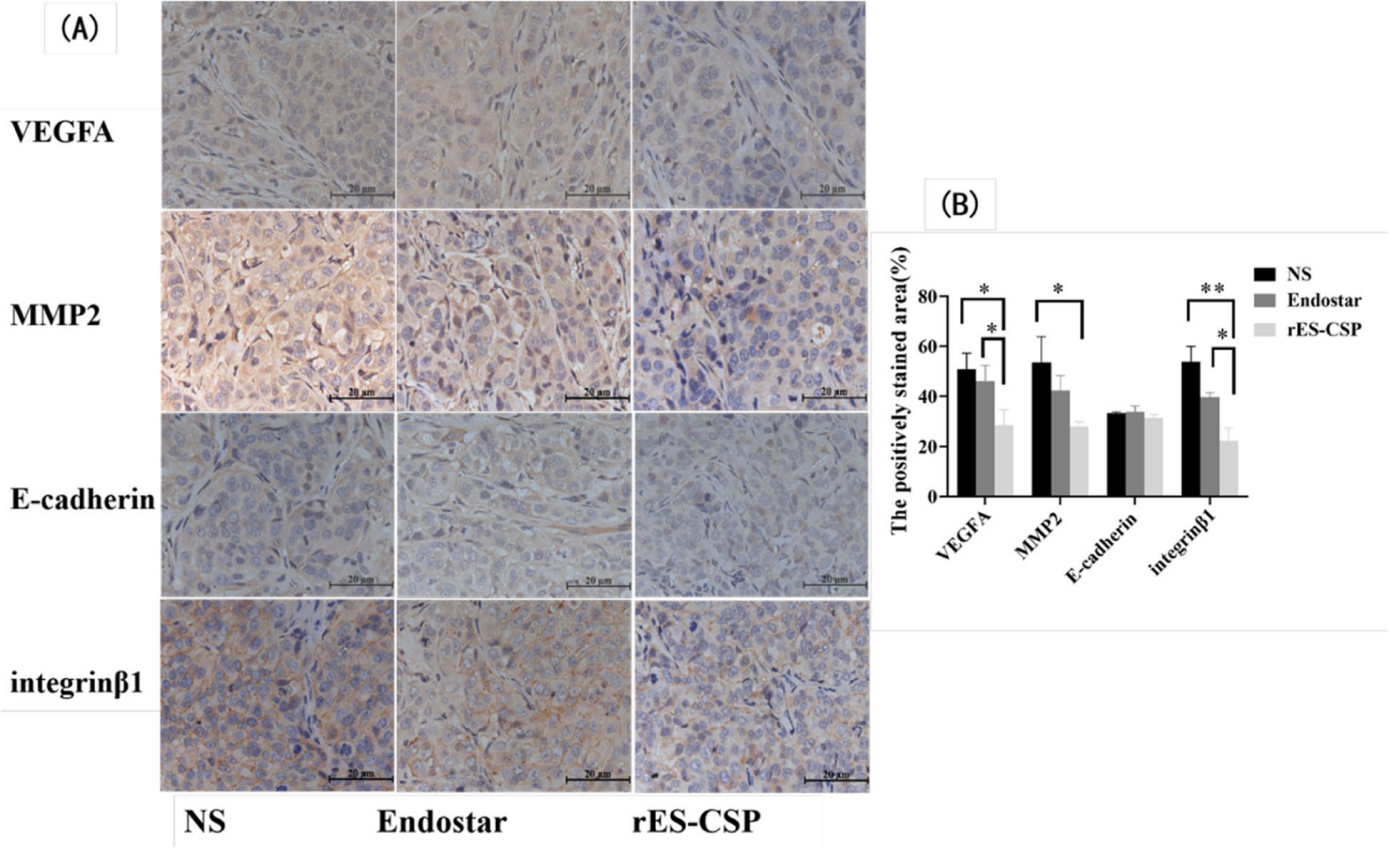
Immunohistochemical staining in orthotopic xenograft tumor. **A** Protein expression of angiogenic factor VEGFA and metastasis-associated molecule. **B** Statistical analysis of the percentage of the positively stained area. rES-CSP group vs NS group; rES-CSP group vs Endostar group; *P < 0.05, **P < 0.01; *n* = 3

Immunohistochemical staining showed that the expression of angiogenic factor VEGFA and integrinβ1 metastasis-associated molecule in rES-CSP group was significantly lower than that in the NS and the Endostar (*P* < 0.05), and the expression of MMP2 and E-cadherin protein was insignificantly changed (Fig. 6). The results indicated that rES-CSP could inhibit the expression of VEGF and integrinβ1 protein in an orthotopic xenograft tumor. Which were consistent with in vitro experiments.

## Discussion

Over the past few years, a series of molecular biomarkers targeting signaling cascades in HCC progression or therapeutic significance have been identified, none has any positive clinical results [23, 24]. A soluble form of recombinant endostatin is available from a yeast system or *Escherichia coli*, which has antiangiogenic and antitumor activities in vivo [25]. A growing body of literature indicates that endostatin gene therapy can inhibit the growth of primary tumors, the development of lung metastases, and angiogenesis in murine models [26–29]. Endostatin could directly bind ovarian cancer cells and inhibit tumor cell attachment and dissemination of ovarian cancer cells, which express significant amounts of alpha (5) beta (1) integrin [30]. Further, Endostatin helps adhesion and migration of murine lung cancer (Lewis lung cancer [LLC]) cells in an integrin-dependent manner, did not demonstrate the functions against the other murine lung cancer cell line, that lacks alpha5beta(1) integrin [31].

RGD (Arg-Gly-Asp) is a good ligand for several integrins including avb3, which is inducible and highly expressed on activated endothelial cells on new blood vessels during angiogenesis. The RGD (CRGDKGPDC; iRGD) modified endostatin, which showed specific and increased binding to endothelial cells was more effective than human endostatin in inhibiting liver cancer growth in athymic mice [32]. The circumsporozoite protein CSP region I-plus (CSP I-plus) binds to the highly-sulfated heparan sulfate proteoglycans (HSPG’s) overexpressed in liver and hepatocellular carcinoma. Our previous study suggested that CSP I-plus modified recombinant human Endostatin (rEndostatin, Endostar) could specifically target to hepatocellular carcinoma cell line HepG2 and made a direct inhibition on tumor cells in vitro. Furthermore, CSP I-plus modified rEndostatin (rES-CSP) also improved the accumulation in liver and HCC tissue, and signifcantly enhanced the inhibitory effects on tumor growth in nude mice with subcutaneous and orthotopic xenograft models of hepatocellular carcinoma HepG2 [21]. In the present study, CSP I-plus modified rEndostatin (rES-CSP) not only inhibited the migration, invasion of high metastatic hepatocellular carcinoma cell line HCCLM3 in vitro, but also suppressed tumorigenesis and tumor metastasis in nude mice with orthotopic xenograft model of hepatocellular carcinoma HCCLM3-Luc. Specifically, HCCLM3-Luc was used in orthotopic xenografts mouse model, which has high metastatic potential with a spontaneous pulmonary metastasis, is labelled with a stable luciferase activity, and is effective in resolving and quantifying the dynamics of tumourigenesis and metastasis though in vivo imaging system. Further, tumor weight and volume, the lung metastasis rate in the rES-CSP group were significantly different compared with the parent Endostar group and the control group.

Metastasis is a nonlinear (i.e., generally irreversible) and dynamic process involving cancer cell motility, intravasation, transit in the blood or lymph, extravasation, and growth at a new site [33]. Apart from distinct cells contribute to the HCC metastasis, many kinds of noncellular components of tumor microenvironment have participated in the tumor metastasis, such as TGF-b [34], vascular endothelial growth factor [35, 36], epidermal growth factor [37], and MMPs [38, 39], E-cadherin [40], so on. The most common hepatocellular carcinoma (HCC) metastases site is lung [41, 42]. Endostatin inhibited local invasion and tumor vascularization of transplanted murine malignant keratinocytes, and reduced the development of highly vascularized murine mammary tumors, at least in part, was associated with its ability to down-regulate VEGF expression within the tumor [43]. Endostatin could co-localizes to tropomysin microfilaments and acts to inhibit AIDS-related Kaposi’s sarcoma (KS) cell migration and invasion in response to the clinically relevant angiogenic cytokines VEGF and bFGF [44]. In addition, endostatin blocks the activation and activities of certain tumor-associated pro-MMPs, such as pro-MMP-2, − 9, and − 13, which may explain, at least in part, the antitumor effect of endostatin [45]. In the study, CSP I-plus modified rEndostatin (rES-CSP) down-regulated the expression of vascular endothelial growth factor VEGFA and metastasis-associated molecule integerβ1 in HCCLM3 and nude mice with orthotopic xenograft model of hepatocellular carcinoma HCCLM3-Luc. Next, HCCLM3 were treated with heparin sodium, which can bind to HSPGs on hepatoma cells and block the binding of CSP I-plus to the surface of hepatoma cells, and analyzed the expression both mRNA and protein levels of VEGFA and MMP2, integerβ1, E-cadherin. Heparin sodium treatment reversed both mRNA and protein levels of VEGFA and MMP2, integerβ1 in HCCLM3, which was down-regulated by rES-CSP. Those revealed that the CSP I-plus could potentially enhance the antitumor effect of Endostar, at least in part, enabled Endostar to bind to the HSPGs receptor in hepatocytes and HCC tissue, and interfered with the function of HSPGs receptors on tumor growth and metastasis, which is consistent with the results of previous studies.

In summary, rES-CSP could inhibit the metastasis of hepatocellular carcinoma in vitro and in vivo, and it was initially determined that rES-CSP may play a critical role in inhibiting tumor growth and metastasis by down-regulating the expression of angiogenesis factor VEGFA and metastasis-associated molecular integrinβ1. It may also play a role in inhibiting hepatocellular carcinoma metastasis by interfering with HSPG receptor-mediated tumor migration. This study provides ideas for drug development to treat hepatocellular carcinoma metastasis, but its clear mechanism needed further research, such as Epithelial-Mesenchymal Transition (EMT), which is important for metastasis.

## Supplementary Information

**Additional file 1: Supplementary Figures**. The full-length blots of WB in Fig. 5B. Whole-cell extracts were resolved on 10% SDS-PAGE by bio-rad Mini-PROTEAN with 10 sample Wells (1Marker, 2space, 3Blank group, 4CSP I-plus group, 5Endostar group, 6rES-CSP group, 7rES-CSP+ heparin group, 8DDP group, 9space, 10Marker), and then electrotransferred onto a polyvinylidene difluoride (PVDF) membrane (Millipore). The membranes were then probed with respective primary antibodies against VEGFA, MMP2, integrinβ1, and E-cadherin procured from Abcam, β-actin from Beyotime Biotechnology, Shanghai, China. Before The membranes were were probed with primary antibodies, the lane of Marker (the sample of Well1 and Well10) was cut off, and the band of target protein was split up in accordance with the expected molecular weight of diffrent primary antibodies. In addition, the data of DDP group wasn’t shown in the manuscript. **Figure S1.** The PVDF membrane was probed with primary antibodies against VEGFA (ab52917, MW 27KDa). **Figure S2.** The PVDF membrane was probed with primary antibodies against β-actin (AA128, MW 43KDa), which was the reference protein of VEGFA in the same gel. **Figure S3.** The PVDF membrane was probed with primary antibodies against MMP2 (ab37150, MW 72KDa). **Figure S4.** The PVDF membrane was probed with primary antibodies against β-actin (AA128, MW 43KDa), which was the reference protein of MMP2 in the same gel. **Figure S5.** The PVDF membrane was probed with primary antibodies agains E-cadherin (ab76055, MW 97KDa)**. Figure S6.** The PVDF membrane was probed with primary antibodies against β-actin (AA128, MW 43KDa), which was the reference protein of E-cadherin in the same gel. **Figure S7.** The PVDF membrane was probed with primary antibodies agains integrin β 1 (ab3039, MW 130KDa). **Figure S8.** The PVDF membrane was probed with primary antibodies against β-actin (AA128, MW 43KDa), which was the reference protein of integrin β 1 in the same gel. (From left to right, 1Marker, 2space, 3Blank group, 4CSP I-plus group, 5Endostar group, 6rES-CSP group, 7rES-CSP+ heparin group, 8DDP group, 9space, 10Marker, respectively).

## Data Availability

The datasets generated and/or analyzed during the study are available from the corresponding author on reasonable request.
