# Supplementary figures and images for: CSP I-plus modified rEndostatin inhibits hepatocellular carcinoma metastasis via down-regulation of VEGFA and integrinβ1
Source: BMC Cancer. 2022 Nov 22;22:1200. doi: 10.1186/s12885-022-10318-8 (PMC9682839; doi:10.1186/s12885-022-10318-8)

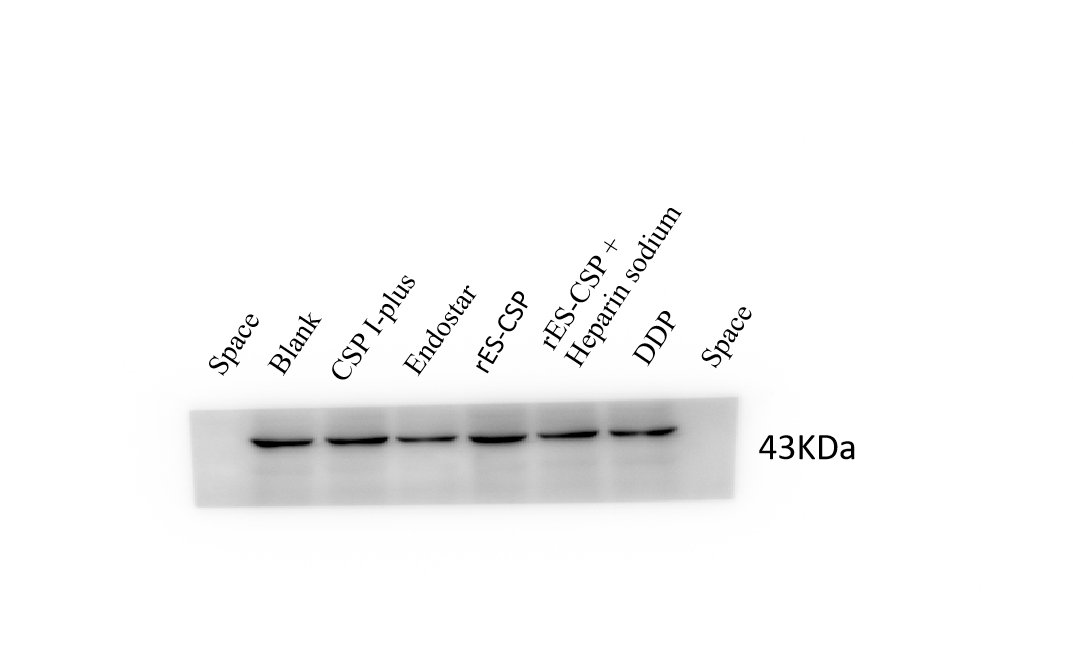

Supplement: Supplementary file 1 — Additional file 1: Supplementary Figures. The full-length blots of WB in Fig. 5B. Whole-cell extracts were resolved on 10% SDS-PAGE by bio-rad Mini-PROTEAN with 10 sample Wells (1Marker, 2space, 3Blank group, 4CSP I-plus group, 5Endostar group, 6rES-CSP group, 7rES-CSP+ heparin group, 8DDP group, 9space, 10Marker), and then electrotransferred onto a polyvinylidene difluoride (PVDF) membrane (Millipore). The membranes were then probed with respective primary antibodies against VEGFA, MMP2, integrinβ1, and E-cadherin procured from Abcam, β-actin from Beyotime Biotechnology, Shanghai, China. Before The membranes were were probed with primary antibodies, the lane of Marker (the sample of Well1 and Well10) was cut off, and the band of target protein was split up in accordance with the expected molecular weight of diffrent primary antibodies. In addition, the data of DDP group wasn’t shown in the manuscript. Figure S1. The PVDF membrane was probed with primary antibodies against VEGFA (ab52917, MW 27KDa). Figure S2. The PVDF membrane was probed with primary antibodies against β-actin (AA128, MW 43KDa), which was the reference protein of VEGFA in the same gel. Figure S3. The PVDF membrane was probed with primary antibodies against MMP2 (ab37150, MW 72KDa). Figure S4. The PVDF membrane was probed with primary antibodies against β-actin (AA128, MW 43KDa), which was the reference protein of MMP2 in the same gel. Figure S5. The PVDF membrane was probed with primary antibodies agains E-cadherin (ab76055, MW 97KDa). Figure S6. The PVDF membrane was probed with primary antibodies against β-actin (AA128, MW 43KDa), which was the reference protein of E-cadherin in the same gel. Figure S7. The PVDF membrane was probed with primary antibodies agains integrin β 1 (ab3039, MW 130KDa). Figure S8. The PVDF membrane was probed with primary antibodies against β-actin (AA128, MW 43KDa), which was the reference protein of integrin β 1 in the same gel. (From left to right, 1Marker, 2space, 3B [file 12885_2022_10318_MOESM1_ESM.zip › Figure S2.tif]

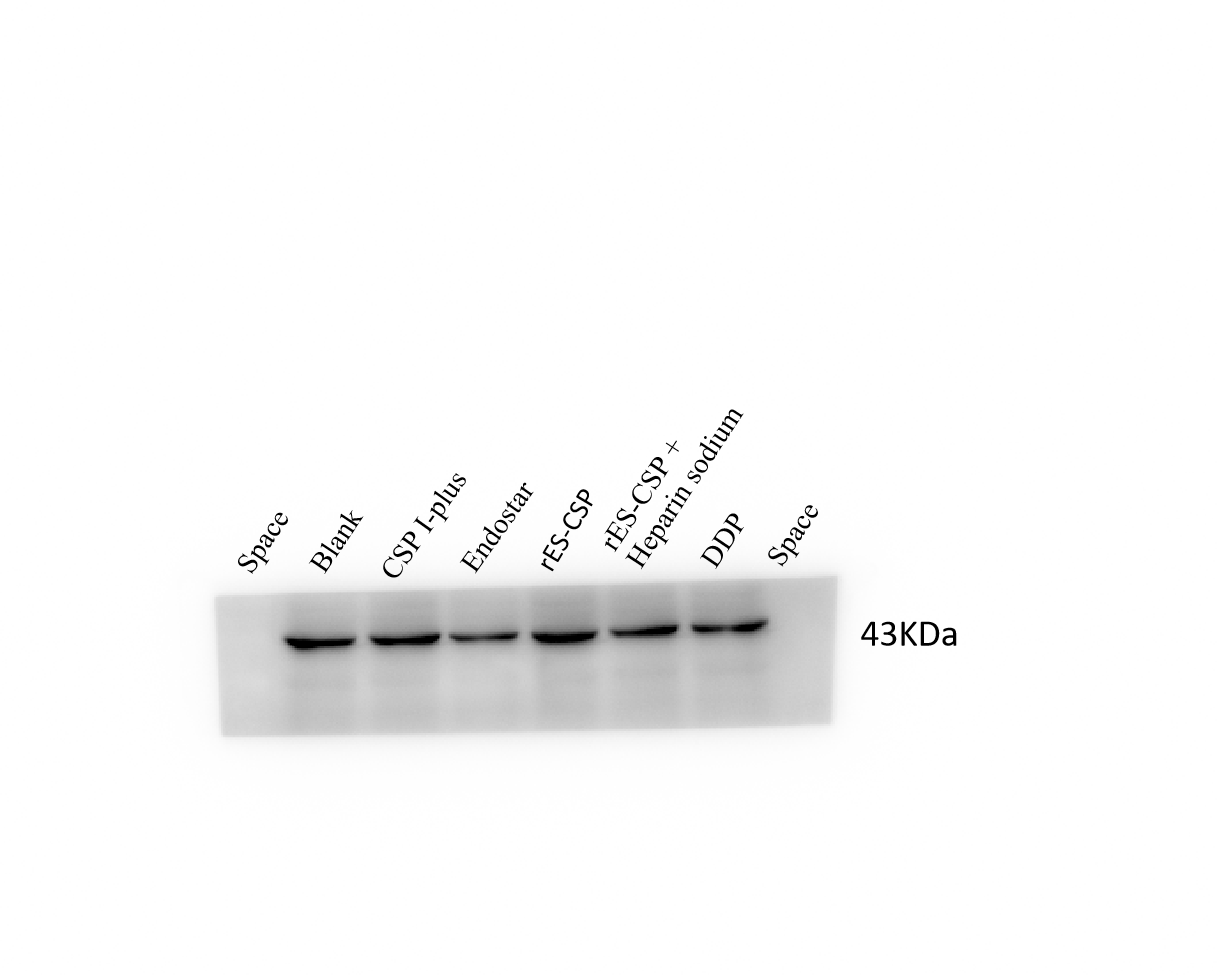

Supplement: Supplementary file 1 — Additional file 1: Supplementary Figures. The full-length blots of WB in Fig. 5B. Whole-cell extracts were resolved on 10% SDS-PAGE by bio-rad Mini-PROTEAN with 10 sample Wells (1Marker, 2space, 3Blank group, 4CSP I-plus group, 5Endostar group, 6rES-CSP group, 7rES-CSP+ heparin group, 8DDP group, 9space, 10Marker), and then electrotransferred onto a polyvinylidene difluoride (PVDF) membrane (Millipore). The membranes were then probed with respective primary antibodies against VEGFA, MMP2, integrinβ1, and E-cadherin procured from Abcam, β-actin from Beyotime Biotechnology, Shanghai, China. Before The membranes were were probed with primary antibodies, the lane of Marker (the sample of Well1 and Well10) was cut off, and the band of target protein was split up in accordance with the expected molecular weight of diffrent primary antibodies. In addition, the data of DDP group wasn’t shown in the manuscript. Figure S1. The PVDF membrane was probed with primary antibodies against VEGFA (ab52917, MW 27KDa). Figure S2. The PVDF membrane was probed with primary antibodies against β-actin (AA128, MW 43KDa), which was the reference protein of VEGFA in the same gel. Figure S3. The PVDF membrane was probed with primary antibodies against MMP2 (ab37150, MW 72KDa). Figure S4. The PVDF membrane was probed with primary antibodies against β-actin (AA128, MW 43KDa), which was the reference protein of MMP2 in the same gel. Figure S5. The PVDF membrane was probed with primary antibodies agains E-cadherin (ab76055, MW 97KDa). Figure S6. The PVDF membrane was probed with primary antibodies against β-actin (AA128, MW 43KDa), which was the reference protein of E-cadherin in the same gel. Figure S7. The PVDF membrane was probed with primary antibodies agains integrin β 1 (ab3039, MW 130KDa). Figure S8. The PVDF membrane was probed with primary antibodies against β-actin (AA128, MW 43KDa), which was the reference protein of integrin β 1 in the same gel. (From left to right, 1Marker, 2space, 3B [file 12885_2022_10318_MOESM1_ESM.zip › Figure S6.tif]

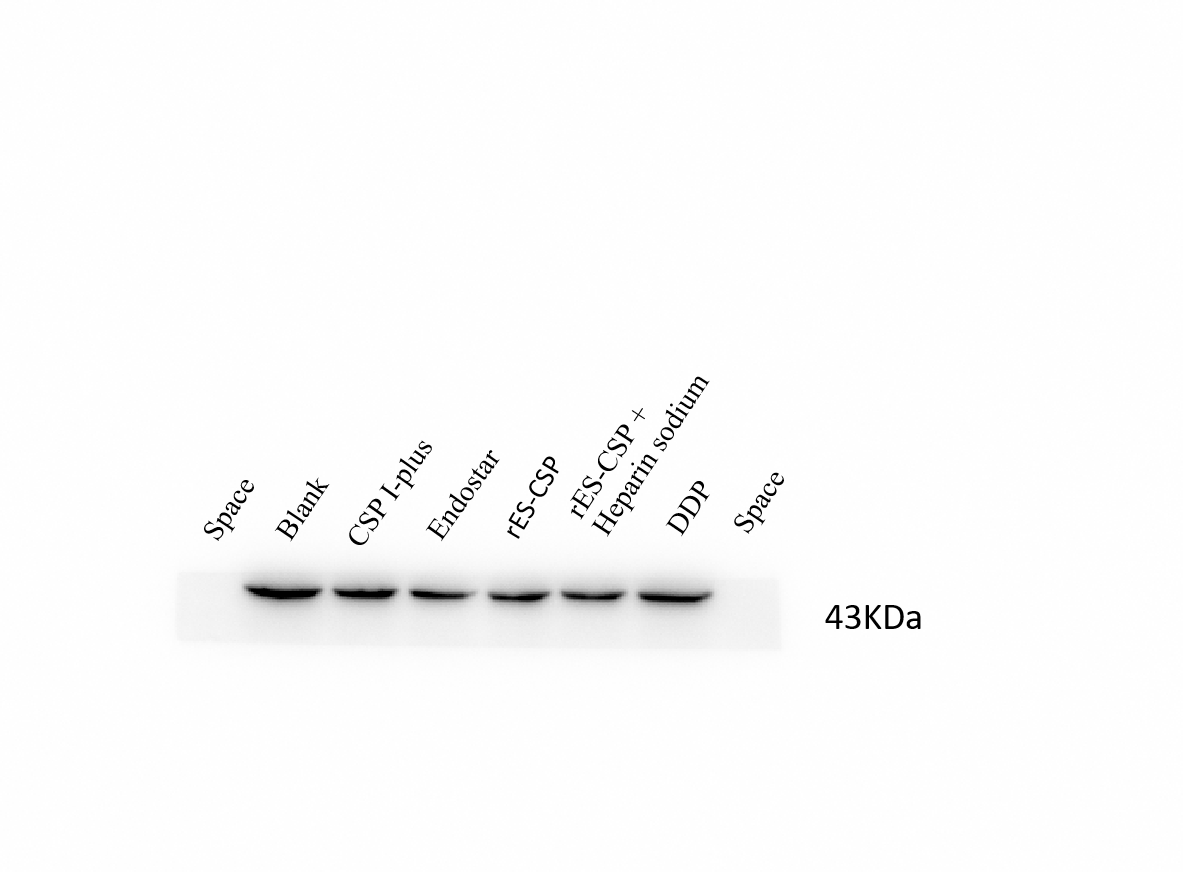

Supplement: Supplementary file 1 — Additional file 1: Supplementary Figures. The full-length blots of WB in Fig. 5B. Whole-cell extracts were resolved on 10% SDS-PAGE by bio-rad Mini-PROTEAN with 10 sample Wells (1Marker, 2space, 3Blank group, 4CSP I-plus group, 5Endostar group, 6rES-CSP group, 7rES-CSP+ heparin group, 8DDP group, 9space, 10Marker), and then electrotransferred onto a polyvinylidene difluoride (PVDF) membrane (Millipore). The membranes were then probed with respective primary antibodies against VEGFA, MMP2, integrinβ1, and E-cadherin procured from Abcam, β-actin from Beyotime Biotechnology, Shanghai, China. Before The membranes were were probed with primary antibodies, the lane of Marker (the sample of Well1 and Well10) was cut off, and the band of target protein was split up in accordance with the expected molecular weight of diffrent primary antibodies. In addition, the data of DDP group wasn’t shown in the manuscript. Figure S1. The PVDF membrane was probed with primary antibodies against VEGFA (ab52917, MW 27KDa). Figure S2. The PVDF membrane was probed with primary antibodies against β-actin (AA128, MW 43KDa), which was the reference protein of VEGFA in the same gel. Figure S3. The PVDF membrane was probed with primary antibodies against MMP2 (ab37150, MW 72KDa). Figure S4. The PVDF membrane was probed with primary antibodies against β-actin (AA128, MW 43KDa), which was the reference protein of MMP2 in the same gel. Figure S5. The PVDF membrane was probed with primary antibodies agains E-cadherin (ab76055, MW 97KDa). Figure S6. The PVDF membrane was probed with primary antibodies against β-actin (AA128, MW 43KDa), which was the reference protein of E-cadherin in the same gel. Figure S7. The PVDF membrane was probed with primary antibodies agains integrin β 1 (ab3039, MW 130KDa). Figure S8. The PVDF membrane was probed with primary antibodies against β-actin (AA128, MW 43KDa), which was the reference protein of integrin β 1 in the same gel. (From left to right, 1Marker, 2space, 3B [file 12885_2022_10318_MOESM1_ESM.zip › Figure S8.tif]

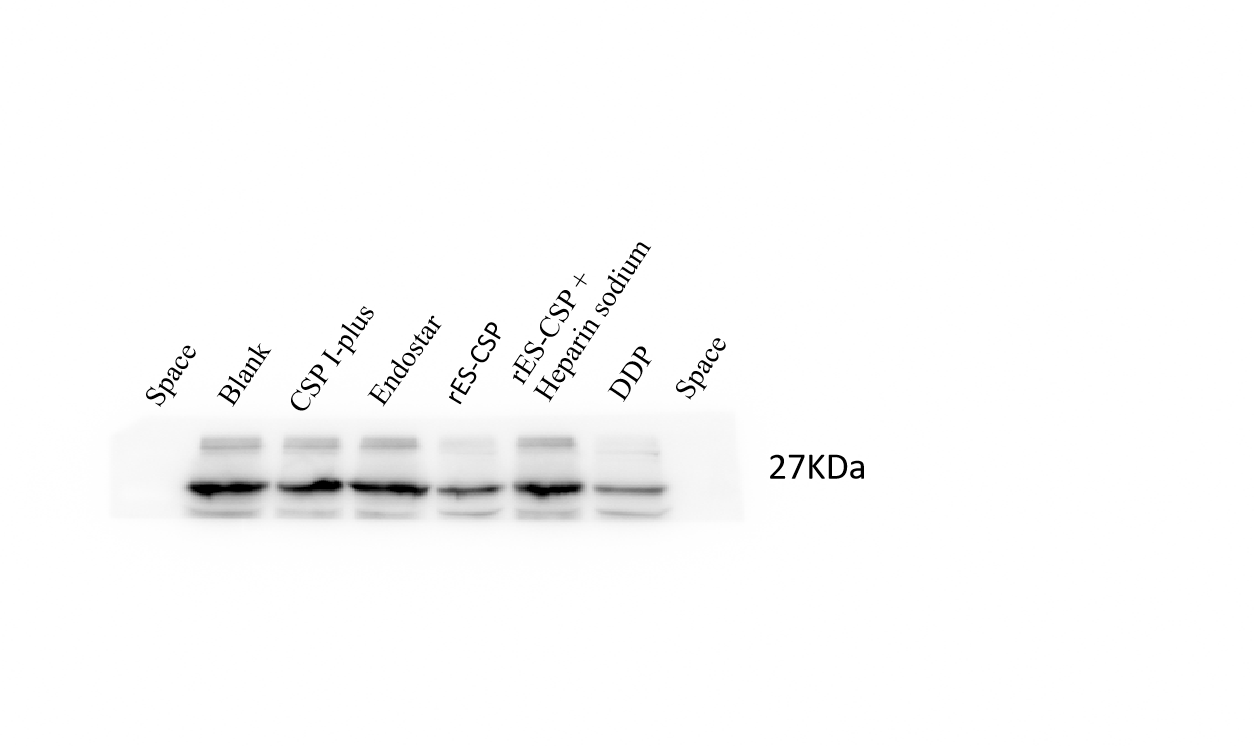

Supplement: Supplementary file 1 — Additional file 1: Supplementary Figures. The full-length blots of WB in Fig. 5B. Whole-cell extracts were resolved on 10% SDS-PAGE by bio-rad Mini-PROTEAN with 10 sample Wells (1Marker, 2space, 3Blank group, 4CSP I-plus group, 5Endostar group, 6rES-CSP group, 7rES-CSP+ heparin group, 8DDP group, 9space, 10Marker), and then electrotransferred onto a polyvinylidene difluoride (PVDF) membrane (Millipore). The membranes were then probed with respective primary antibodies against VEGFA, MMP2, integrinβ1, and E-cadherin procured from Abcam, β-actin from Beyotime Biotechnology, Shanghai, China. Before The membranes were were probed with primary antibodies, the lane of Marker (the sample of Well1 and Well10) was cut off, and the band of target protein was split up in accordance with the expected molecular weight of diffrent primary antibodies. In addition, the data of DDP group wasn’t shown in the manuscript. Figure S1. The PVDF membrane was probed with primary antibodies against VEGFA (ab52917, MW 27KDa). Figure S2. The PVDF membrane was probed with primary antibodies against β-actin (AA128, MW 43KDa), which was the reference protein of VEGFA in the same gel. Figure S3. The PVDF membrane was probed with primary antibodies against MMP2 (ab37150, MW 72KDa). Figure S4. The PVDF membrane was probed with primary antibodies against β-actin (AA128, MW 43KDa), which was the reference protein of MMP2 in the same gel. Figure S5. The PVDF membrane was probed with primary antibodies agains E-cadherin (ab76055, MW 97KDa). Figure S6. The PVDF membrane was probed with primary antibodies against β-actin (AA128, MW 43KDa), which was the reference protein of E-cadherin in the same gel. Figure S7. The PVDF membrane was probed with primary antibodies agains integrin β 1 (ab3039, MW 130KDa). Figure S8. The PVDF membrane was probed with primary antibodies against β-actin (AA128, MW 43KDa), which was the reference protein of integrin β 1 in the same gel. (From left to right, 1Marker, 2space, 3B [file 12885_2022_10318_MOESM1_ESM.zip › Figure S1.tif]

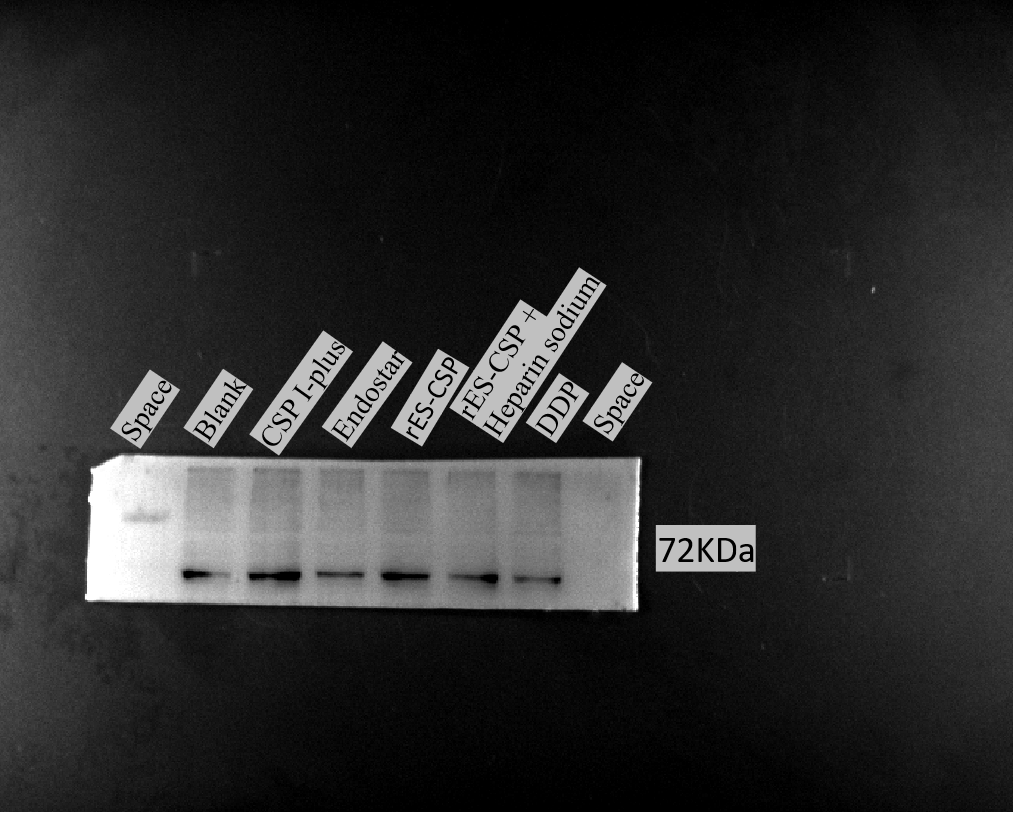

Supplement: Supplementary file 1 — Additional file 1: Supplementary Figures. The full-length blots of WB in Fig. 5B. Whole-cell extracts were resolved on 10% SDS-PAGE by bio-rad Mini-PROTEAN with 10 sample Wells (1Marker, 2space, 3Blank group, 4CSP I-plus group, 5Endostar group, 6rES-CSP group, 7rES-CSP+ heparin group, 8DDP group, 9space, 10Marker), and then electrotransferred onto a polyvinylidene difluoride (PVDF) membrane (Millipore). The membranes were then probed with respective primary antibodies against VEGFA, MMP2, integrinβ1, and E-cadherin procured from Abcam, β-actin from Beyotime Biotechnology, Shanghai, China. Before The membranes were were probed with primary antibodies, the lane of Marker (the sample of Well1 and Well10) was cut off, and the band of target protein was split up in accordance with the expected molecular weight of diffrent primary antibodies. In addition, the data of DDP group wasn’t shown in the manuscript. Figure S1. The PVDF membrane was probed with primary antibodies against VEGFA (ab52917, MW 27KDa). Figure S2. The PVDF membrane was probed with primary antibodies against β-actin (AA128, MW 43KDa), which was the reference protein of VEGFA in the same gel. Figure S3. The PVDF membrane was probed with primary antibodies against MMP2 (ab37150, MW 72KDa). Figure S4. The PVDF membrane was probed with primary antibodies against β-actin (AA128, MW 43KDa), which was the reference protein of MMP2 in the same gel. Figure S5. The PVDF membrane was probed with primary antibodies agains E-cadherin (ab76055, MW 97KDa). Figure S6. The PVDF membrane was probed with primary antibodies against β-actin (AA128, MW 43KDa), which was the reference protein of E-cadherin in the same gel. Figure S7. The PVDF membrane was probed with primary antibodies agains integrin β 1 (ab3039, MW 130KDa). Figure S8. The PVDF membrane was probed with primary antibodies against β-actin (AA128, MW 43KDa), which was the reference protein of integrin β 1 in the same gel. (From left to right, 1Marker, 2space, 3B [file 12885_2022_10318_MOESM1_ESM.zip › Figure S3.tif]

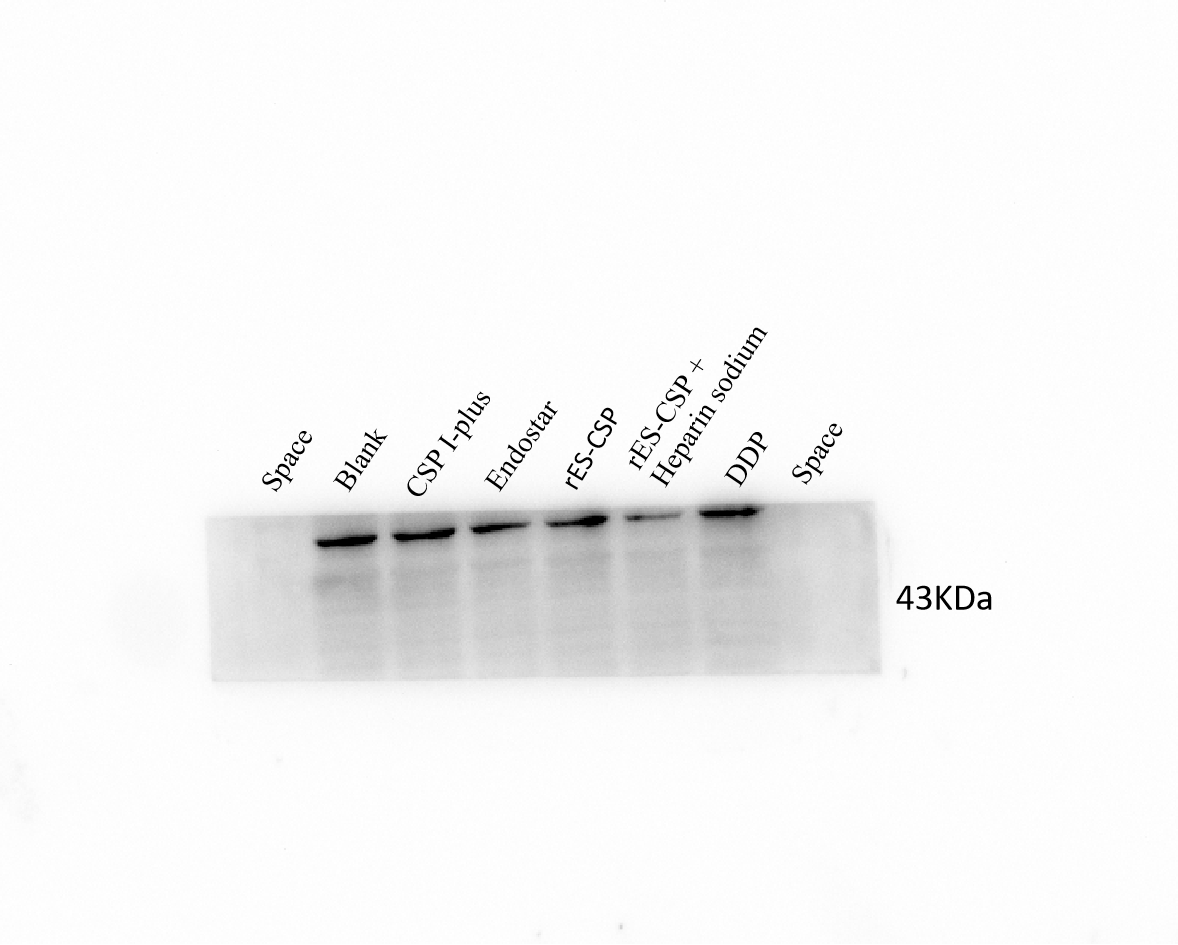

Supplement: Supplementary file 1 — Additional file 1: Supplementary Figures. The full-length blots of WB in Fig. 5B. Whole-cell extracts were resolved on 10% SDS-PAGE by bio-rad Mini-PROTEAN with 10 sample Wells (1Marker, 2space, 3Blank group, 4CSP I-plus group, 5Endostar group, 6rES-CSP group, 7rES-CSP+ heparin group, 8DDP group, 9space, 10Marker), and then electrotransferred onto a polyvinylidene difluoride (PVDF) membrane (Millipore). The membranes were then probed with respective primary antibodies against VEGFA, MMP2, integrinβ1, and E-cadherin procured from Abcam, β-actin from Beyotime Biotechnology, Shanghai, China. Before The membranes were were probed with primary antibodies, the lane of Marker (the sample of Well1 and Well10) was cut off, and the band of target protein was split up in accordance with the expected molecular weight of diffrent primary antibodies. In addition, the data of DDP group wasn’t shown in the manuscript. Figure S1. The PVDF membrane was probed with primary antibodies against VEGFA (ab52917, MW 27KDa). Figure S2. The PVDF membrane was probed with primary antibodies against β-actin (AA128, MW 43KDa), which was the reference protein of VEGFA in the same gel. Figure S3. The PVDF membrane was probed with primary antibodies against MMP2 (ab37150, MW 72KDa). Figure S4. The PVDF membrane was probed with primary antibodies against β-actin (AA128, MW 43KDa), which was the reference protein of MMP2 in the same gel. Figure S5. The PVDF membrane was probed with primary antibodies agains E-cadherin (ab76055, MW 97KDa). Figure S6. The PVDF membrane was probed with primary antibodies against β-actin (AA128, MW 43KDa), which was the reference protein of E-cadherin in the same gel. Figure S7. The PVDF membrane was probed with primary antibodies agains integrin β 1 (ab3039, MW 130KDa). Figure S8. The PVDF membrane was probed with primary antibodies against β-actin (AA128, MW 43KDa), which was the reference protein of integrin β 1 in the same gel. (From left to right, 1Marker, 2space, 3B [file 12885_2022_10318_MOESM1_ESM.zip › Figure S4.tif]

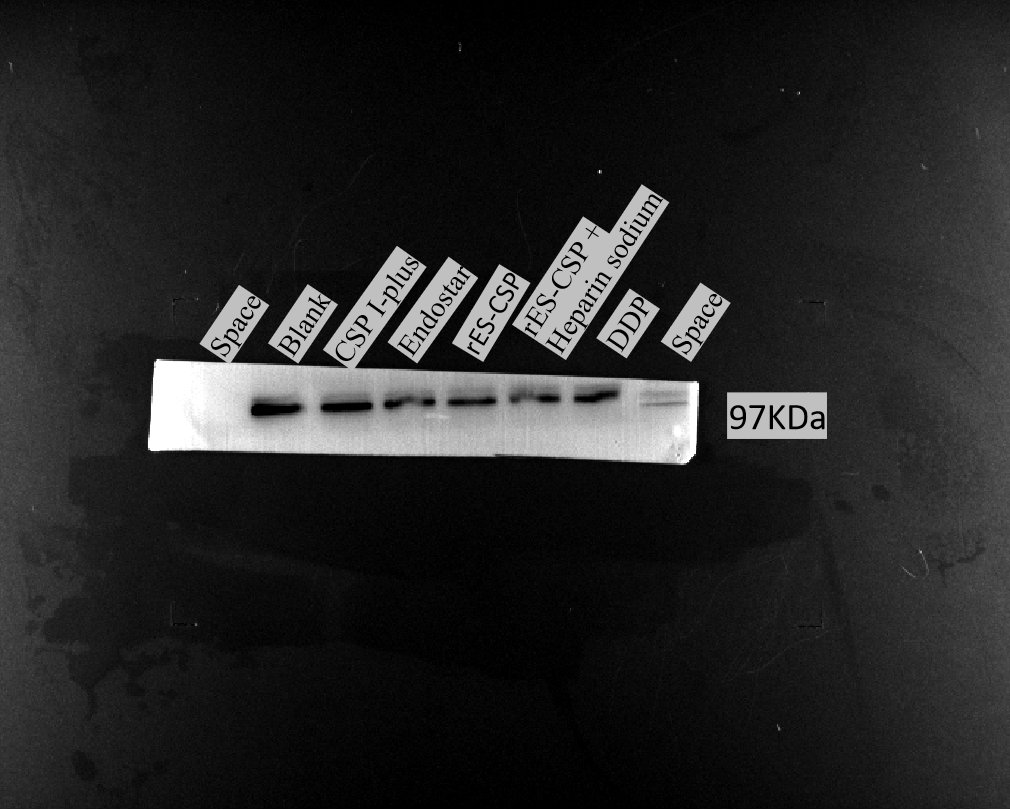

Supplement: Supplementary file 1 — Additional file 1: Supplementary Figures. The full-length blots of WB in Fig. 5B. Whole-cell extracts were resolved on 10% SDS-PAGE by bio-rad Mini-PROTEAN with 10 sample Wells (1Marker, 2space, 3Blank group, 4CSP I-plus group, 5Endostar group, 6rES-CSP group, 7rES-CSP+ heparin group, 8DDP group, 9space, 10Marker), and then electrotransferred onto a polyvinylidene difluoride (PVDF) membrane (Millipore). The membranes were then probed with respective primary antibodies against VEGFA, MMP2, integrinβ1, and E-cadherin procured from Abcam, β-actin from Beyotime Biotechnology, Shanghai, China. Before The membranes were were probed with primary antibodies, the lane of Marker (the sample of Well1 and Well10) was cut off, and the band of target protein was split up in accordance with the expected molecular weight of diffrent primary antibodies. In addition, the data of DDP group wasn’t shown in the manuscript. Figure S1. The PVDF membrane was probed with primary antibodies against VEGFA (ab52917, MW 27KDa). Figure S2. The PVDF membrane was probed with primary antibodies against β-actin (AA128, MW 43KDa), which was the reference protein of VEGFA in the same gel. Figure S3. The PVDF membrane was probed with primary antibodies against MMP2 (ab37150, MW 72KDa). Figure S4. The PVDF membrane was probed with primary antibodies against β-actin (AA128, MW 43KDa), which was the reference protein of MMP2 in the same gel. Figure S5. The PVDF membrane was probed with primary antibodies agains E-cadherin (ab76055, MW 97KDa). Figure S6. The PVDF membrane was probed with primary antibodies against β-actin (AA128, MW 43KDa), which was the reference protein of E-cadherin in the same gel. Figure S7. The PVDF membrane was probed with primary antibodies agains integrin β 1 (ab3039, MW 130KDa). Figure S8. The PVDF membrane was probed with primary antibodies against β-actin (AA128, MW 43KDa), which was the reference protein of integrin β 1 in the same gel. (From left to right, 1Marker, 2space, 3B [file 12885_2022_10318_MOESM1_ESM.zip › Figure S5.tif]

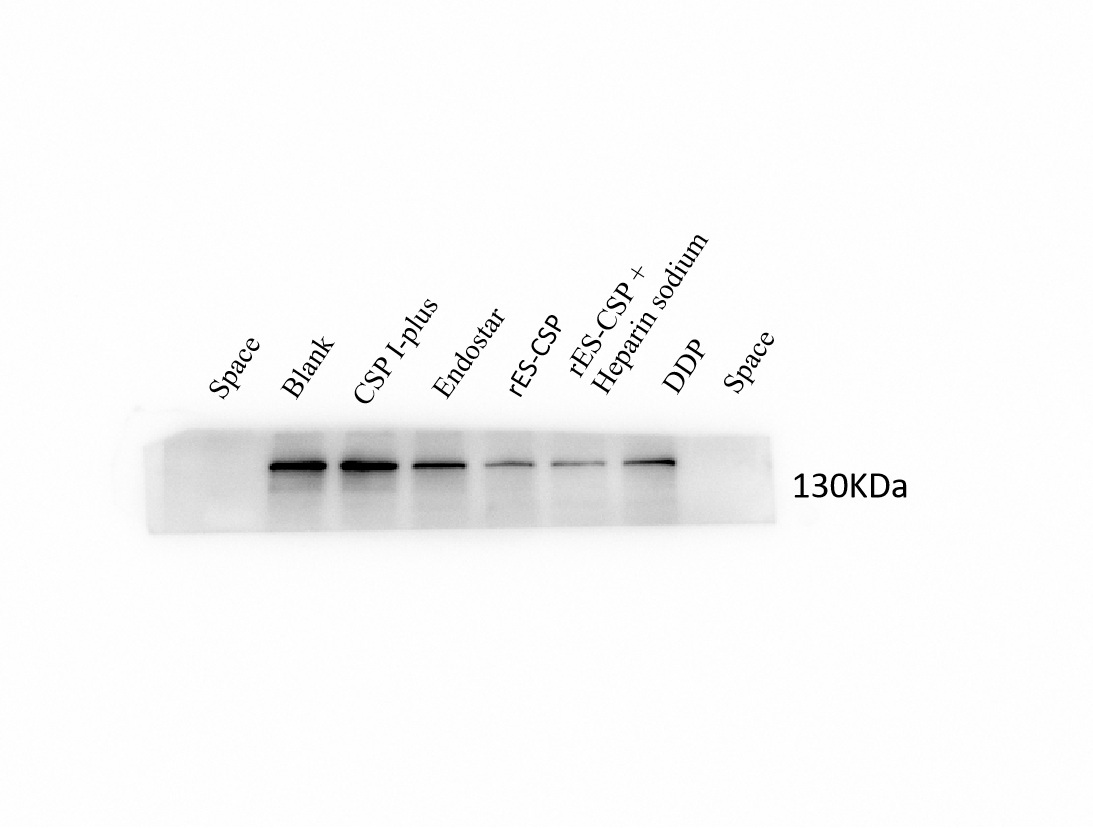

Supplement: Supplementary file 1 — Additional file 1: Supplementary Figures. The full-length blots of WB in Fig. 5B. Whole-cell extracts were resolved on 10% SDS-PAGE by bio-rad Mini-PROTEAN with 10 sample Wells (1Marker, 2space, 3Blank group, 4CSP I-plus group, 5Endostar group, 6rES-CSP group, 7rES-CSP+ heparin group, 8DDP group, 9space, 10Marker), and then electrotransferred onto a polyvinylidene difluoride (PVDF) membrane (Millipore). The membranes were then probed with respective primary antibodies against VEGFA, MMP2, integrinβ1, and E-cadherin procured from Abcam, β-actin from Beyotime Biotechnology, Shanghai, China. Before The membranes were were probed with primary antibodies, the lane of Marker (the sample of Well1 and Well10) was cut off, and the band of target protein was split up in accordance with the expected molecular weight of diffrent primary antibodies. In addition, the data of DDP group wasn’t shown in the manuscript. Figure S1. The PVDF membrane was probed with primary antibodies against VEGFA (ab52917, MW 27KDa). Figure S2. The PVDF membrane was probed with primary antibodies against β-actin (AA128, MW 43KDa), which was the reference protein of VEGFA in the same gel. Figure S3. The PVDF membrane was probed with primary antibodies against MMP2 (ab37150, MW 72KDa). Figure S4. The PVDF membrane was probed with primary antibodies against β-actin (AA128, MW 43KDa), which was the reference protein of MMP2 in the same gel. Figure S5. The PVDF membrane was probed with primary antibodies agains E-cadherin (ab76055, MW 97KDa). Figure S6. The PVDF membrane was probed with primary antibodies against β-actin (AA128, MW 43KDa), which was the reference protein of E-cadherin in the same gel. Figure S7. The PVDF membrane was probed with primary antibodies agains integrin β 1 (ab3039, MW 130KDa). Figure S8. The PVDF membrane was probed with primary antibodies against β-actin (AA128, MW 43KDa), which was the reference protein of integrin β 1 in the same gel. (From left to right, 1Marker, 2space, 3B [file 12885_2022_10318_MOESM1_ESM.zip › Figure S7.tif]
